# The Proline 7 Substitution in the Preproneuropeptide Y Is Associated with Higher Hepatic Lipase Activity In Vivo

**DOI:** 10.1155/2017/2869090

**Published:** 2017-05-30

**Authors:** Stephan Schiekofer, Marcus E. Kleber, Winfried Maerz, Franz M. Rasche, Jochen G. Schneider

**Affiliations:** ^1^Geriatric Medicine, University of Regensburg, Regensburg, Germany; ^2^Vth Department of Medicine, Mannheim Medical Faculty, Heidelberg University, Mannheim, Germany; ^3^Clinical Institute of Medical and Chemical Laboratory Diagnostics, Medical University Graz, Graz, Austria; ^4^Synlab Academy, Mannheim, Germany; ^5^Synlab Holding Deutschland GmbH, Augsburg, Germany; ^6^Departments of Internal Medicine, Neurology, Dermatology, Clinic for Endocrinology, Diabetology, and Nephrology, Section of Nephrology, University Leipzig, Leipzig, Germany; ^7^Luxembourg Centre for Systems Biomedicine (LCSB), University of Luxembourg and Centre Hospitalier Emile Mayrisch (CHEM), Esch-sur-Alzette, Luxembourg; ^8^Department of Internal Medicine II, Saarland University Medical Center, Homburg, Saar, Germany

## Abstract

Hepatic lipase (HL) functions as a lipolytic enzyme that hydrolyzes triglycerides and phospholipids present in circulating plasma lipoproteins. Plasma HL activity is known to be regulated by hormonal and metabolic factors, but HL responsiveness to insulin as well as its role in modulating atherosclerotic risk is still controversial. We investigated on the influence of a known polymorphism in the neurotransmitter neuropeptide Y (NPY) on HL activity in two different cohorts consisting of diabetic and nondiabetic patients. HL activity was 24% and 34% higher on nondiabetic and diabetic subjects in the presence of the 7Pro allele in NPY, respectively. The presence of the 7Pro allele was an independent predictor of HL activity in multivariate analyses in both cohorts. These data suggest a regulatory effect of NPY on HL activity. Among carriers of the 7Pro allele, we also found a statistically significant lower absolute number of infarctions compared to noncarriers (*p* < 0.05) and a nonsignificant trend towards less myocardial infarction in the 7Pro allele diabetic carriers (*p* = 0.085). In conclusion, the common 7Pro allele in NPY was associated with higher HL activity in nondiabetic and diabetic subjects and its presence seems to coincide with a lower frequency of certain cardiovascular events.

## 1. Introduction

Hepatic lipase (HL) is a glycoprotein mainly, but not exclusively, secreted by hepatocytes and bound to heparan sulfate proteoglycans at the surface of the liver sinusoidal capillaries [1, 2]. HL plays a key role in the metabolism of lipoproteins as it hydrolyzes triglycerides and phospholipids of LDL and HDL cholesterols. It is thereby involved in the formation of atherogenic small dense LDL particles from larger, buoyant LDL particles and represents a major determinant of plasma HDL concentration [3, 4]. The influence of HL activity on HDL cholesterol and the generation of small dense LDL cholesterol imply a role for HL in atherosclerosis. Yet, there is no consensus as to whether HL effects are primarily pro- or antiatherogenic [5–7].

HL is predominantly regulated directly and/or indirectly by cell cholesterol content on a transcriptional level [8], probably involving a sterol response element in its promoter region [9]. It is also regulated by several hormonal and metabolic factors such as glucocorticoids, estrogen, thyroid hormones, and adrenalin (as reviewed by Perret el al. [10]). Insulin is also an important activator of HL activity in vivo. Insulin levels do correlate positively with HL activity [10], and insulin directly increases HL activity in vivo [11]. Consequently, it has been reported that HL is increased in insulin resistance (IR) [12] and in type 2 diabetes [13], although the exact mechanism on how HL activity changes in these situations is still controversial [11, 14]. Meanwhile, it has been assumed that increased HL activity causes a drop in HDL concentrations and promotes the formation of small dense LDL particles in insulin-resistant states [12]. More recently, HL activity has been set in context with hepatic steatosis and nonalcoholic fatty liver disease (NAFLD) and its consequences [15].

NPY is a sympathetic neurotransmitter that is widely expressed in the peripheral and central nervous system. Central NPY is known to affect body weight by the regulation of food intake and satiety [16]. Previous studies have demonstrated an effect of a T1128C single nucleotide polymorphism (Leu7Pro) in the signal peptide of NPY (prepro-NPY) on parameters of lipid metabolism, glucose control, and even vascular disease: The 7Pro substitution has been associated with higher total [17] and LDL cholesterol levels [18], increased blood pressure [19], increased risk for type 2 diabetes [20], the frequency of the metabolic syndrome [21, 22], and increased vascular disease [19, 23–27]. Other reports have shown that, in contrast, the 7Pro allele is associated with enhanced endothelium-dependent vascular dilatation [28] and consequently decreased coronary artery disease [29]. Because the 7Pro substitution has no direct effect on plasma NPY levels [30], it is still unclear to date how the Leu7Pro variant affects peripheral metabolic parameter, such as cholesterol, and vascular disease. HL is a major determinant of cholesterol metabolism and is also involved in vascular disease. Therefore, we hypothesized that there could be an association between the Leu7Pro polymorphism in NPY and HL activities as a potential mechanism on how the Leu7Pro in prepro-NPY would influence lipid levels or diseases.

The aim of this study was to test whether the Leu7Pro substitution in NPY is associated with HL activity in subjects with or without type 2 diabetes mellitus.

## 2. Research Design and Methods

### 2.1. Study Subjects

We studied two different cohorts of patients. At first, 210 consecutive male patients with diagnosed or suspected coronary artery disease (CAD) were studied. All of these individuals underwent elective coronary angiography and were referred to as nondiabetic CAD. Of those, 170 had a severe score of >1. The overt diagnosis of diabetes, according to the American Diabetes Association criteria (fasting plasma glucose > 126 mg/dL), was an exclusion criterion in this group. Among the CAD patients, 71 were treated with and 89 without a statin.

Furthermore, 91 male patients with known type 2 diabetes mellitus were studied. The diabetic patients enrolled in the study were treated by either dietary intervention or oral antidiabetic drugs (sulfonylurea drugs, metformin, acarbose, glinides, or combinations). 25 subjects were treated with insulin and were omitted from the homeostasis model assessment (HOMA) calculation. In both groups, heparin therapy in the previous 72 h, contraindications for heparin administration, severe kidney or liver disease, treatment with drugs known to affect HL plasma levels, and a fasting triglyceride level > 1000 mg/dL, suggesting secondary lipid disorders, were the exclusion criteria.

All patients were advised not to consume alcohol and to maintain a standard diet composed of about 25% protein, 15% fat, and 60% carbohydrates during the study period. They were also advised not to eat or drink from 10 pm on the day before the study visit. Concomitant medication as well as antidiabetic medication (in the diabetes group) was maintained at a constant dose during the study period. The study was approved by the Internal Ethics Committee of Heidelberg University, and each patient gave informed consent.

### 2.2. Hepatic Lipase

EDTA blood was obtained 10 min after a bolus injection of 60 IU heparin/kg/body weight (Braun Melsungen, Melsungen, Germany) after an overnight fast. Postheparin HL activity (nmol/mL/min) was determined in duplicates using a triolein/phosphatidylcholine emulsion as described previously [31]. The samples were immediately chilled to 4°C, centrifuged, and stored at −80°C until assayed. The samples were quantitated in duplicates, and postheparin plasma from pooled normal control subjects was used to correct for interassay variation. The intraassay coefficient was 8%, and the interassay coefficient was 11%.

### 2.3. Analysis of Other Biochemical Variables

Fasting venous blood was sampled for the measurement of plasma concentrations of glucose, serum lipids, and insulin. Plasma glucose was measured by a glucose oxidase method. Insulin samples were quantitated in duplicates by ELISA and immunoreactivity, respectively (B-Bridge International Inc., San Jose, USA and Cisbio International, F91192 Gif-Sur-Yvette Cedex, France). The degree of insulin resistance (HOMA) was estimated according to the method described by Matthews et al. [32].

Total cholesterol, HDL cholesterol, and triglyceride concentrations were enzymatically determined with a Synchron LX-20 (Beckman Coulter, Munich, Germany). LDL and VLDL were separated by ultracentrifugation in a Beckman LM-8 ultracentrifuge in 100 *μ*l volumes with a VT-51.2 rotor (Beckman Coulter).

### 2.4. Genotyping

The preproneuropeptide Y genotypes were determined using a previously described method [18].

### 2.5. Statistical Analyses

Statistical analyses were performed with SPSS for Windows, release 15.0 (SPSS Inc., Chicago, USA). Comparisons of quantitative variables between the two sets of patients were performed by Student's *t*-test after log-transformation (HL activity) or Mann–Whitney *U* test as appropriate. Two-tailed bivariate correlations were determined by the Pearson coefficient for parameters with normal distribution and with the Spearman coefficient for parameters with other distributions. Multiple linear regression analysis was used to identify independent correlations, using HL as dependent and included age, BMI, plasma triglycerides, VLDL-C, HDL-C, HOMA-IR, and NPY genotype at the same time as independent variables. The cumulative event frequency of categorical variables (genotype and myocardial infarction) was assessed by contingency tables and appropriate testing (Fisher's exact test, chi-square test). Results are expressed as mean ± SD. A *p* value of less than 0.05 was considered statistically significant.

## 3. Results

### 3.1. Association of the 7Pro Allele with Higher HL Activity in Male Nondiabetic Subjects

**Figure 1:**
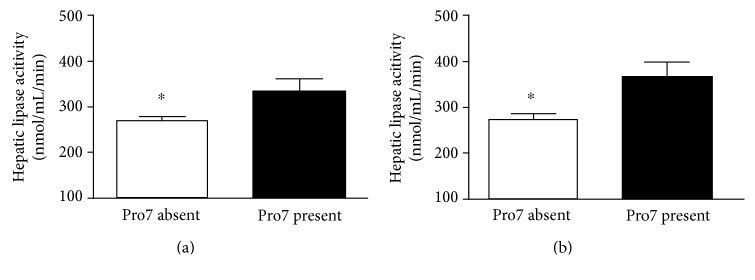
HL activity (mean ± SEM) in postheparin plasma in the absence or presence of the 7Pro allele in preproneuropetide Y for (a) nondiabetic male patients and (b) male diabetic patients.

The Leu7Pro polymorphism was detected with an allele frequency of 0.05 in the nondiabetic patients which is in within the range of earlier publications. Genotypes were consistent with the Hardy-Weinberg equilibrium (chi-square value 0.64, *p* = 0.4). Among the subjects within this group, no homozygous carrier of the 7Pro allele was identified.  Table 1 shows the anthropometric and metabolic parameters of the 210 male patients according to the NPY genotype. 7Pro was associated with a significant 24% higher HL activity (270 ± 98 versus 335 ± 106 nmol/mL/min, *p* < 0.01, Figure 1(a)). We have not detected any other significant association between the Leu7Pro polymorphism and other metabolic parameters in the nondiabetic male patients. HL activity was significantly positively correlated with insulin levels (*r* = 0.146, *p* = 0.05) and negatively with HDL cholesterol (*r* = −0.145, *p* < 0.05) in this group. In a multivariate regression analysis, controlling for BMI, age, and markers of lipid and glucose metabolism as known confounding factors that may influence HL activity, the presence of 7Pro was an independent predictor for HL activity together with HDL cholesterol (Table 2). The significant association of HL activity with insulin was lost in the regression analysis. There was no collinearity between the Leu7Pro polymorphism and HDL levels regarding HL activity, indicating that both parameters may be independently associated with HL activity.

### 3.2. Association of the 7Pro Allele with Higher HL Activity in Male Diabetic Patients

To confirm the association of the Leu7Pro polymorphism in NPY with HL activity in a separate group of patients, we assessed the NPY polymorphism in 91 male patients with type 2 diabetes of which HL activity levels were available. Their anthropometric data are shown in Table 3. The frequency of the 7Pro allele in the diabetic patients was 0.04, which was not significantly different from the observed frequency in the nondiabetic patients (0.05 and 0.04, *p* = 0.83, Fisher's test). The genotypes were within the Hardy-Weinberg equilibrium (chi-square value 0.22, *p* = 0.66). In the diabetes group, we did also not find a homozygous carrier of the 7Pro allele. The metabolic parameters in the diabetes group were not different between carriers and noncarriers of the 7Pro allele apart from glucose metabolism (Table 3), but we found a significant 34% increase in HL activity, carriers of the 7Pro allele versus noncarriers (277 ± 113 versus 373 ± 80 nmol/mL/min, *p* < 0.05, Figure 1(b)). Simple correlation coefficients showed a significant negative association between HL activity and fasting plasma glucose levels (*r* = −0.248, *p* < 0.05) and age (*r* = −0.223, *p* < 0.05). In the multivariate regression analysis with HL activity as dependent and distinct parameters of glucose and lipid metabolism as potential confounding controls, the 7Pro allele and age remained the only significant predictors for HL activity (Table 2). The positive association of HL activity with glucose levels was not confirmed in the regression analysis, and, different from the nondiabetic males, HDL cholesterol was not correlated with HL activity in this study group. Both the 7Pro allele and age were independently influencing HL activity.

### 3.3. Leu7Pro Substitution and LPL Activity

There was no significant influence of the 7Pro allele on LPL activity in either group, although we did observe a nonsignificant trend towards a lower LPL activity in diabetic carriers of the 7Pro allele.

### 3.4. Leu7Pro Substitution and Vascular Disease Parameters

The Leu7Pro substitution had been implicated in vascular disease in previous studies. In this regard, we found a significantly simple inverse correlation between the presence of the 7Pro allele and report of myocardial infarction in the medical history in the nondiabetic CAD subjects (*r* = −0.15, *p* < 0.05). The absolute number of infarctions was statistically lower among carriers of 7Pro allele as compared to the noncarriers (see Supplemental Table 1 available online at https://doi.org/10.1155/2017/2869090). This observation was not accompanied by a significant association between the presence of the 7Pro allele and any available quantitative parameter of coronary disease in the nondiabetic CAD patients (extent score *r* = −0.077, *p* = 0.291, severe score *r* = −0.05, *p* = 0.47). Nevertheless, we recorded a nonsignificant trend towards lower number of 7Pro carriers in higher extent score quartiles (*r* = −0.113, *p* = 0.11).

Similar to the nondiabetic CAD patients, there was an inverse, but nonsignificant trend between the history of myocardial infarction and the 7Pro allele in diabetic men (*r* = −0.2, *p* = 0.085). There was a tendency towards lower coronary disease history reports in the presence of the 7Pro allele (*r* = −0.16, *p* = 0.19). In this group, 5 out of 8 carriers of the 7Pro allele were reported to have suffered from myocardial infarction versus 37 out of 83 noncarriers (*p* = 0.6, chi-square test).

## 4. Discussion

Our results show significantly (24%) higher HL activity in nondiabetic subjects that carry the 7Pro allele as compared to the wild type. Surprisingly, we did not find any other significant association between the 7Pro allele of the NPY polymorphism and other metabolic parameter (Table 1). This observation is in contrast to several other reports, where associations between 7Pro and lipid parameters were found to be significant [17, 18]. To confirm the observation on the effect of the Leu7Pro polymorphism on HL activity, we studied the finding in a separate group of male patients with type 2 diabetes mellitus. Male diabetic patients with the 7Pro substitution had even a higher increase in HL activity (34%) versus the noncarriers. Again, no other significant association existed in the diabetic patients.

**Figure 2:**
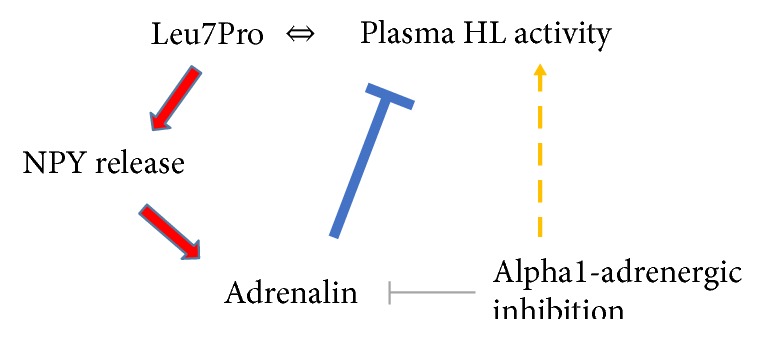
Sketch depicting the potential association between the Leu7Pro polymorphism and plasma HL activity: the presence of the SNP modifies the stress-induced release of NPY and factors of the sympathetic nervous system that are coregulated. Sympathetic action blocks HL maturation and secretion from the liver cells. HL secretion is indirectly stimulated by alpha1B-adrenergic inhibition.

Our observation might be puzzling for a moment. The 7Pro substitution has also no direct effect on plasma NPY levels [25]. However, as neurotransmitter NPY is simultaneously stored and released with noradrenaline and adrenalin [33, 34], it is released from the sympathetic nerves and in the adrenals upon stress. Moreover, NPY has a regulatory role on overall catecholamine synthesis and secretion [35]. Carriers of the 7Pro allele were found to have a decreased basal but enhanced peripheral NPY release upon sympathetic stimulation [36, 37]. Catecholamines on the other hand as well as alpha-adrenergic stimulation have an inhibitory effect on HL secretion, and inhibition of alpha-adrenergic signalling stimulates the release of HL from hepatocytes [38–40]. These data lead us to speculate that the link between NPY and HL might be the peripheral sympathetic system (Figure 2). Our data are of pure observational nature and thus, further proof of this hypothesis must come from follow-up studies that include the assessment of HL activity and mass simultaneously. The exact reason for the mostly isolated but confirmed increase in HL activity in the presence of the 7Pro allele remains unclear.

Our results are insofar surprising as the influence of the 7Pro substitution on lipid parameters was previously reported, and this fact was at least part of the motive for our study [17, 18]. Gender and diet can be ruled out as confounders as we decided to exclusively study male subjects, and all participants were advised to maintain a stable diet during the study period. In addition, locoregional differences may exist as the 7Pro allele in NPY was detected at higher frequency in Finns compared to Dutchmen, and its impact on serum cholesterol concentration seemed to be stronger in obese than that in normal-weight subjects [18]. As expected, we found HDL cholesterol levels as independent factor to influence HL activity in addition to the 7Pro allele. However, we observed no collinearity between any lipoprotein parameter and the polymorphism in NPY in our study regarding their association with HL activity. In diabetic subjects, the known association between HDL cholesterol and HL [41] activity was missing. This phenomenon might be caused by a strong regulatory effect of glucose metabolism on HL activity [14], reflected by the correlation between fasting glucose and HL activity in the diabetic subjects. This observation was not sustained in linear regression possibly due to the influence of the IR-modifying therapies as well as various degrees of hepatic insulin resistance.

Our results may also not turn the balance on the previous studies providing arguments for both beneficial [29] and detrimental contributions of the 7Pro allele with regard to vascular disease [19, 23, 24]. The Leu7Pro polymorphism correlated inversely with the presence of CAD and the occurrence of myocardial infarction in our cohorts, although these associations were of modest significance. The at-best mild effects of the 7Pro allele on CAD may be blurred by the missing associations with the plasma lipoproteins and IR that have been demonstrated in other studies [16, 18]. In addition, HL activity would probably better be accompanied by dynamic assessments of lipoproteins or subgroup analyses within the endogenous pathways rather than static measures to find an association. Hence, our data do come probably too far afield to allow a speculation on the role of HL in atherosclerosis, a subject that very recently gained novel attraction [42].

In summary, higher HL activity was detected in carriers of the 7Pro allele in two independent cohorts of men with and without diabetes mellitus independently of lipoprotein concentrations and markers of glucose metabolism. These findings allow the speculation that the Leu7Pro polymorphism in NPY may exert a regulatory effect on HL activity.

## Supplementary Material

Supplemental Table 1 Contingency table for the history of myocardial infarction according to the NPY genotype in non-diabetic male CAD patients.

## Figures and Tables

**Table 1:** Anthropometric and biochemical variables of the 210 male study subjects.

Variable	7Pro absent	7Pro present	*p*
*N*	188	22	
Age (years)	61.8 ± 9.3	60.0 ± 9.6	0.30
BMI (kg/m^2^)	27.5 ± 3.3	28.0 ± 3.5	0.49
Total cholesterol (mg/dL)	205.2 ± 47.3	210.3 ± 42.2	0.61
LDL cholesterol (mg/dL)	140.3 ± 39.4	146.6 ± 38.2	0.48
HDL cholesterol (mg/dL)	39.5 ± 10.8	39.8 ± 9.5	0.9
VLDL cholesterol (mg/dL)	26.3 ± 33.3	23.9 ± 12.8	0.75
Triglycerides (mg/dL)	154.3 ± 125.7	153.7 ± 80.4	0.97
Insulin (pmol/L)	23.8 ± 11.3	23.06 ± 9.05	0.90
HOMA	6.8 ± 5.11	6.1 ± 3.0	0.93

Data are means ± SD.

**Table 2:** Multiple regression analysis result of variables with significant effect on hepatic lipase (HL) activity.

Independent variable^∗^	Nondiabetic subjects		Diabetic subjects	
	*T*	*β*	*T*	*β*
Age	−0.37	−0.03	−2.25	−0.23^#^
BMI	0.34	0.031	−1.31	−0.14
HDL	−2.64	−0.21^∗^	−0.71	−0.07
Total cholesterol	0.092	0.017	0.206	0.022
Insulin	1.70	0.43	0.03	0.16
HOMA	−1.04	−0.34	0.575	0.063
NPY	2.4	0.21^‡^	2.4	0.25^#^

The dependent variable is hepatic lipase (nanomoles per milliliter per minute). *β* is the standardized coefficient, and *T* represents the estimated coefficient, divided by its own standard error. *T* values below −2 or above 2 are considered as useful predictors in the model.

^∗^ *p* = 0.009; ^‡^*p* = 0.018; ^#^*p* < 0.05.

**Table 3:** Anthropometric and biochemical variables of the 91 male diabetic subjects.

Variable	7Pro absent	7Pro present	*p*
*N*	83	8	
Age (years)	54.6 ± 9.0	51.5 ± 4.9	0.63
BMI (kg/m^2^)	28.6 ± 4.2	30.3 ± 5.3	0.45
Total cholesterol (mg/dL)	215.0 ± 54.0	227.8 ± 46.8	0.57
LDL cholesterol (mg/dL)	138.5 ± 38.6	142.5 ± 42.6	0.80
HDL cholesterol (mg/dL)	36.7 ± 11.0	31.6 ± 10.9	0.31
VLDL cholesterol (mg/dL)	45.9 ± 62.3	52.5 ± 32.9	0.80
Triglycerides (mg/dL)	214.0 ± 214.8	299.5 ± 161.9	0.34
Fasting plasma glucose (mg/dL)	153.9 ± 43.5	196.3 ± 74.4	0.42
Insulin (pmol/L)	43.4 ± 15	37.7 ± 27.38	0.75
HbA_1c_ (%)	7.46 ± 1.0	7.45 ± 0.5	0.45

Data are means ± SD.
