# Supplementary material for: The Proline 7 Substitution in the Preproneuropeptide Y Is Associated with Higher Hepatic Lipase Activity In Vivo
Source: Int J Endocrinol. 2017 May 30;2017:2869090. doi: 10.1155/2017/2869090 (PMC5468775; doi:10.1155/2017/2869090)
Supplement: Supplementary file 1 — Supplemental Table 1 Contingency table for the history of myocardial infarction according to the NPY genotype in non-diabetic male CAD patients. [file 2869090.f1.docx]

Supplemental Table 1 Contingency table for the history of myocardial infarction according to the NPY genotype in non-diabetic male CAD patients

|  | NPY genotype | |  |
| --- | --- | --- | --- |
|  | 7Pro absent | 7Pro present | Total number |
| Infarction not reported | 106 | 17 | 123 |
| Infarction reported | 81 | 4 | 85 |
| Total number | 187 | 21 | 208 |

*P<0.05*

(2 subjects were omitted from analysis because no information about myocardial infarction in past medical history could be obtained)
